# Enrichment and detection of bone disseminated tumor cells in models of low tumor burden

**DOI:** 10.1038/s41598-018-32653-2

**Published:** 2018-09-24

**Authors:** Miranda E. Sowder, Rachelle W. Johnson

**Affiliations:** 1 Program in Cancer Biology, Vanderbilt University, Nashville, TN 37232 USA; 2 Vanderbilt Center for Bone Biology, Department of Medicine, Division of Clinical Pharmacology, Vanderbilt University Medical Center, Nashville, TN 37232 USA; 3 Department of Medicine, Division of Clinical Pharmacology, Vanderbilt University Medical Center, Nashville, TN 37232 USA

## Abstract

Breast cancer cells frequently home to the bone, but the mechanisms controlling tumor colonization of the bone marrow remain unclear. We report significant enrichment of bone-disseminated estrogen receptor positive human MCF7 cells by 17 β-estradiol (E2) following intracardiac inoculation. Using flow cytometric and quantitative PCR approaches, tumor cells were detected in >80% of MCF7 tumor-inoculated mice, regardless of E2, suggesting that E2 is not required for MCF7 dissemination to the bone marrow. Furthermore, we propose two additional models in which to study prolonged latency periods by bone-disseminated tumor cells: murine D2.0R and human SUM159 breast carcinoma cells. Tumor cells were detected in bone marrow of up to 100% of D2.0R and SUM159-inoculated mice depending on the detection method. These findings establish novel models of bone colonization in which to study mechanisms underlying tumor cell seeding to the marrow and prolonged latency, and provide highly sensitive methods to detect these rare events.

## Introduction

Increased morbidity and mortality of breast cancer patients is strongly associated with the development of metastatic lesions by disseminated tumor cells (DTCs). Breast cancer cells frequently metastasize to skeletal sites, where they can cause adverse effects including bone pain, fractures, spinal cord compression, and hypercalcemia^1,2^. Recent evidence, including the detection of DTCs in the bone marrow of patients with early stage breast cancer^3^ and comparative genomic analysis of DTCs and primary tumors^4^, suggests that dissemination of breast cancer cells is an early event. Although systemic adjuvant therapies have improved the relapse-free and overall survival of patients, there is evidence to suggest that DTCs can evade therapy-induced or microenvironment-induced stresses and ultimately evolve into a clinically detectable metastasis^5,6^. A recent meta-analysis of ~63,000 women with estrogen receptor-positive (ER+) breast cancer reported that primary tumor diameter and nodal status, which are indicators of tumor aggressiveness, were most strongly correlated with the risk of distant recurrence^7^. Of particular interest, even patients with no nodal involvement at diagnosis had an appreciable 10–17% risk of developing distant metastasis during years 5–20 after primary diagnosis, suggesting prolonged periods of tumor dormancy. Additionally, approximately 70% of breast cancer patients who succumb to disease have evidence of bone metastasis at autopsy^8,9^. Together, these studies suggest that DTCs may remain in a dormant state for an extended period of time^10^ and that breast cancer survivors are at a significant risk of developing overt bone lesions from DTCs.

Despite the high prevalence of skeletal metastases in breast cancer patients, there are currently no therapeutic options to cure metastatic disease. This deficit is in part due to our limited understanding of the mechanisms that regulate bone colonization and tumor dormancy^11,12^. The identification of factors regulating bone colonization is complicated by the multitude of microenvironmental factors in distant metastatic sites, which differentially affect the homing of DTCs and metastatic progression. Interestingly, several studies have proposed that dormancy-associated factors may act in a tissue-specific manner^13^. In breast cancer, these mechanisms are further complicated by the clinical association of estrogen receptor (ER) status and time to recurrence. At first relapse, skeletal metastases commonly present in ER− breast cancer patients within 5 years of diagnosis; while skeletal recurrence in ER+ breast cancer patients can also present within these first 5 years, the majority of patients recur 8–10 years after diagnosis^14,15^. While differential recurrence patterns between subtypes may not apply to all patients, these clinical observations suggest that there may also be subtype-specific mechanisms underlying tumor cell dormancy and/or reactivation of DTCs in the bone.

A major limitation to studying mechanisms that regulate tumor dormancy and metastatic outgrowth in the bone is the lack of *in vivo* models that recapitulate prolonged tumor latency, as well as our limited ability to detect low levels of tumor burden in bone. Many studies have used the human MDA-MB-231 (ER−) and murine 4T1 (ER−) cells, or sub-clones of these cell lines, but these cell lines are highly aggressive and rapidly induce osteolytic lesions in the bone^16^. We^17^ and others^18,19^ have reported that the human MCF7 (ER+) cell line is non-proliferative in the lung and bone and induces little osteolytic bone destruction, and have proposed this cell line as a clinically relevant model of tumor dormancy. Previous literature reports that MCF7 cells require exogenous 17β-estradiol (E2) to form orthotopic tumors and bone metastases^20,21^; however, E2 results in a dramatic increase in bone volume^22^ and perturbation of normal bone microarchitecture in tumor-inoculated as well as naïve mice. Further, estrogen supplementation causes adverse urinary tract effects resulting in mice being sacrificed before the experimental end-point^20,23^. Importantly, the presence of micrometastatic bone lesions in the absence of E2 has not been rigorously investigated using methods that can detect low tumor burden in the bone.

We report that MCF7 cells are able to colonize the bone marrow following intracardiac inoculation in the presence and absence of E2. Furthermore, we report for the first time that murine D2.0R (ER+) mammary carcinoma and human SUM159 (ER−) breast cancer cells, which have been shown to lie dormant in the lungs following tail vein injection^18,24^, disseminate to the bone marrow with extended latency periods. For the MCF7 and SUM159 models, a highly sensitive and human-specific flow cytometry protocol using CD298 (also known as ATP1B3) expression was implemented, which has been used to identify human breast cancer cells in PDX mice^25^. Further, we capitalized on the human origin of these cells to analyze human versus mouse housekeeping genes by qPCR from whole bone homogenates to quantify tumor burden in bone. In order to detect murine D2.0R cells in the bone marrow, cytokeratin expression was analyzed using immunostaining and qPCR analysis. These highly sensitive methods to detect low metastatic burden are ultimately summarized for their applicability to each tumor model. The proposed techniques to detect small, but significant, changes in metastatic burden, in combination with these novel tumor models, will be instrumental in investigating breast tumor cell homing and extended latency periods in the bone.

## Results

### Establishment of the MCF7, SUM159, and D2.0R timelines

**Figure 1:**
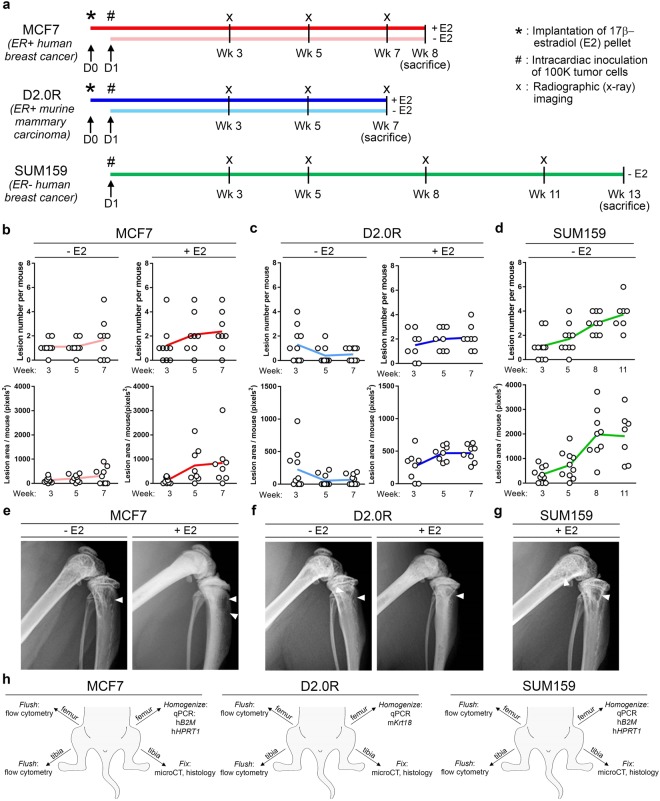
Experimental timeline for MCF7, D2.0R, and SUM159 models and osteolysis. (**a**) Schematic of model timelines from implantation of 17β-estradiol pellets (indicated by asterisk) and inoculation of tumor cells (indicated by pound symbol) to sacrifice. Light colored lines = −E2 mice and dark colored lines = +E2 mice. n = 10 mice inoculated per group. (**b**–**d**) Radiographic assessment of total lesion number per mouse and total lesion area per mouse over time in the (**b**) MCF7 (n = 10 −E2 mice, n = 8 +E2 mice), (**c**) D2.0R (n = 10 –E2 mice, n = 8 +E2 mice) and (**d**) SUM159 models (n = 8 mice). (**e**–**g**) Radiographic images at week 7 for the (**e**) MCF7 and (**f**) D2.0R models and at week 11 for the (**g**) SUM159 models. White arrowheads indicate osteolytic lesions. (**h**) Schematic indicating the methods performed on the hind limbs for each tumor model.

Human MCF7 (ER+) and SUM159 (ER−) tumor cells, and syngeneic murine D2.0R (ER+) cells were inoculated by intracardiac injection. In order to test the estrogen dependence of MCF7 and D2.0R cells in the bone, we implanted a cohort of mice with 17β-estradiol pellets (+E2 mice, dark red and dark blue lines) 24 hours prior to tumor cell intracardiac inoculation while another cohort of mice received no 17β-estradiol pellet (−E2 mice, light red and light blue lines) (Fig. 1a).

Osteolytic bone destruction was monitored *in vivo* by radiography every other week until sacrifice. A gradual increase in lesion number and lesion area was observed by radiography for the −E2 and +E2 MCF7 (Fig. 1b,e), +E2 D2.0R (Fig. 1c,f), and SUM159 (Fig. 1d,g) tumor models throughout the time course. A slight reduction in lesion number and lesion area was observed over time in the −E2 D2.0R tumor model (Fig. 1c). The MCF7 and D2.0R tumor models were sacrificed 7–8 weeks post-inoculation and the SUM159 model 13 weeks after tumor inoculation (Fig. 1a). These timelines were established in order to maintain statistical power for each cohort following several mice becoming moribund or found deceased. Notably, mice in the +E2 cohorts were lost due to the negative urinary tract effects of estradiol supplementation. Mice lost in the D2.0R- and SUM159-inoculated −E2 mice were moribund or found deceased with no evidence of macrometastatic disease or other illness (e.g. infection). Importantly, −E2 and +E2 mice for the MCF7 and D2.0R models were sacrificed at the same time point in order to directly compare tumor burden between the groups. To assess tumor burden in the bone, the hind limbs were dissected at sacrifice and processed for flow cytometry, qPCR, microcomputed tomography (microCT), or histology depending on the tumor model (Fig. 1h).

### E2 enrichment for human tumor cells in the bone marrow by CD298 flow cytometric analysis

For flow cytometry analysis of tumor burden in bone, the human specificity of the CD298 antibody was confirmed by staining non-tumor-inoculated (naïve) mouse bone marrow, which produced no background staining (Supplementary Fig. 1a). Murine D2.0R cells, which do not express human CD298 and therefore serve as an additional negative control, showed no enrichment for human CD298 after staining (Supplementary Fig. 1b). In contrast, >99% of human MCF7 (Supplementary Fig. 1c) and SUM159 (Supplementary Fig. 1c) cell lines were positive for CD298. Human-specific EpCAM and pan-cytokeratin, which are commonly used to detect tumor cells^26,27^, were also tested but resulted in background staining of mouse bone marrow (data not shown). For flow cytometry analysis, gates were established based on staining of non-tumor-inoculated (naïve) mouse bone marrow controls for each experiment and tumor model.

**Figure 2:**
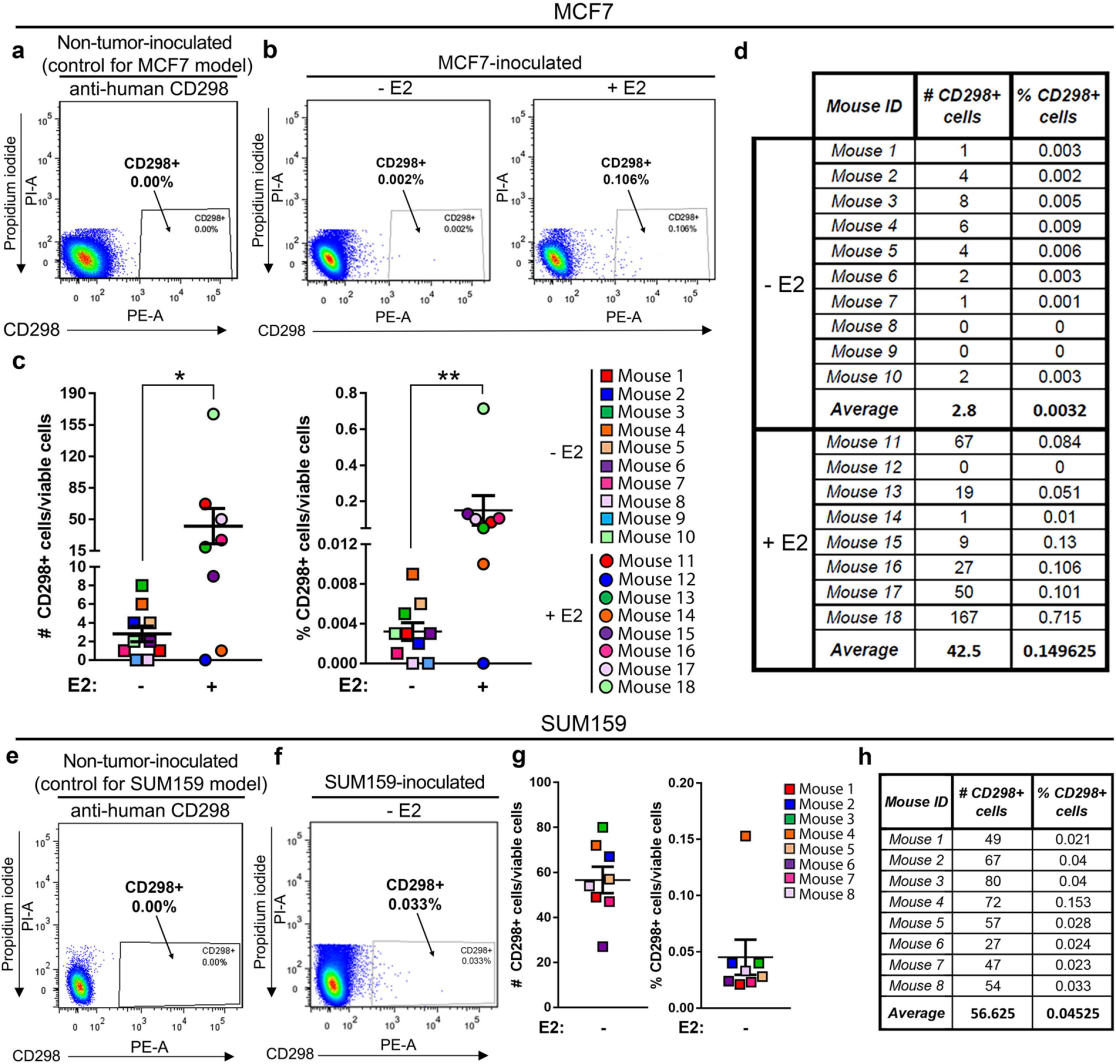
Detection of CD298+ tumor cells in the bone using flow cytometry. (**a**) Representative flow cytometry plot of CD298 staining in non-tumor-inoculated (naïve) mouse bone marrow used as a negative control for the MCF7 model (representative of n = 3 mice). Dead cells (PI+) have been excluded. (**b**) Representative flow cytometry plots of bone marrow from MCF7-inoculated −E2 (n = 10 mice) and +E2 (n = 8 mice) mice. Dead cells (PI+) have been excluded. (**c**,**d**) Quantitation of total number and percent of CD298+ tumor cells from (**b**). (**e**) Representative flow cytometry plot of CD298 staining in non-tumor-inoculated (naïve) mouse bone marrow used as a negative control for the SUM159 model (representative of n = 3 mice). Dead cells (PI+) have been excluded. (**f**) Representative flow cytometry plot of bone marrow from SUM159-inoculated mice (n = 8 mice). Dead cells (PI+) have been excluded. (**g**,**h**) Quantitation of total number and percent of CD298+ tumor cells from (**f**). (**c**) Mann-Whitney U-Test, *P < 0.05 and **P < 0.01.

Staining of non-tumor-inoculated (naïve) mouse bone marrow showed 0% staining for CD298 and was used as a negative control to establish the gates for each experiment and tumor model (Fig. 2a). Bone marrow isolated from mice inoculated with MCF7 cells showed significant enrichment for CD298 staining in mice supplemented with E2 (+E2) by flow cytometry (Fig. 2b) compared to mice that did not receive E2 (−E2). MCF7-inoculated mice showed an average of 2.8 (0.0032%) and 42.5 (0.149%) CD298 + cells in −E2 and +E2 mice, respectively (Fig. 2c,d). By this method, we detected significant enrichment in the number and percent of MCF7 tumor cells in the bones of +E2 mice compared to −E2 mice. Importantly, although the yield of CD298+ tumor cells per mouse was low, MCF7 cells were detected in 8/10 (80%) −E2 and 7/8 (88%) +E2 mice (Fig. 2d). Similarly, compared to non-tumor-inoculated (naïve) control bone marrow (Fig. 2e), which showed 0% staining for CD298, the SUM159 model had detectable tumor cells in 8/8 (100%) mice with an average of 56.6 (0.045%) CD298+ cells (Fig. 2f-h).

### Assessment of E2 effects on MCF7 tumor burden in bone

**Figure 3:**
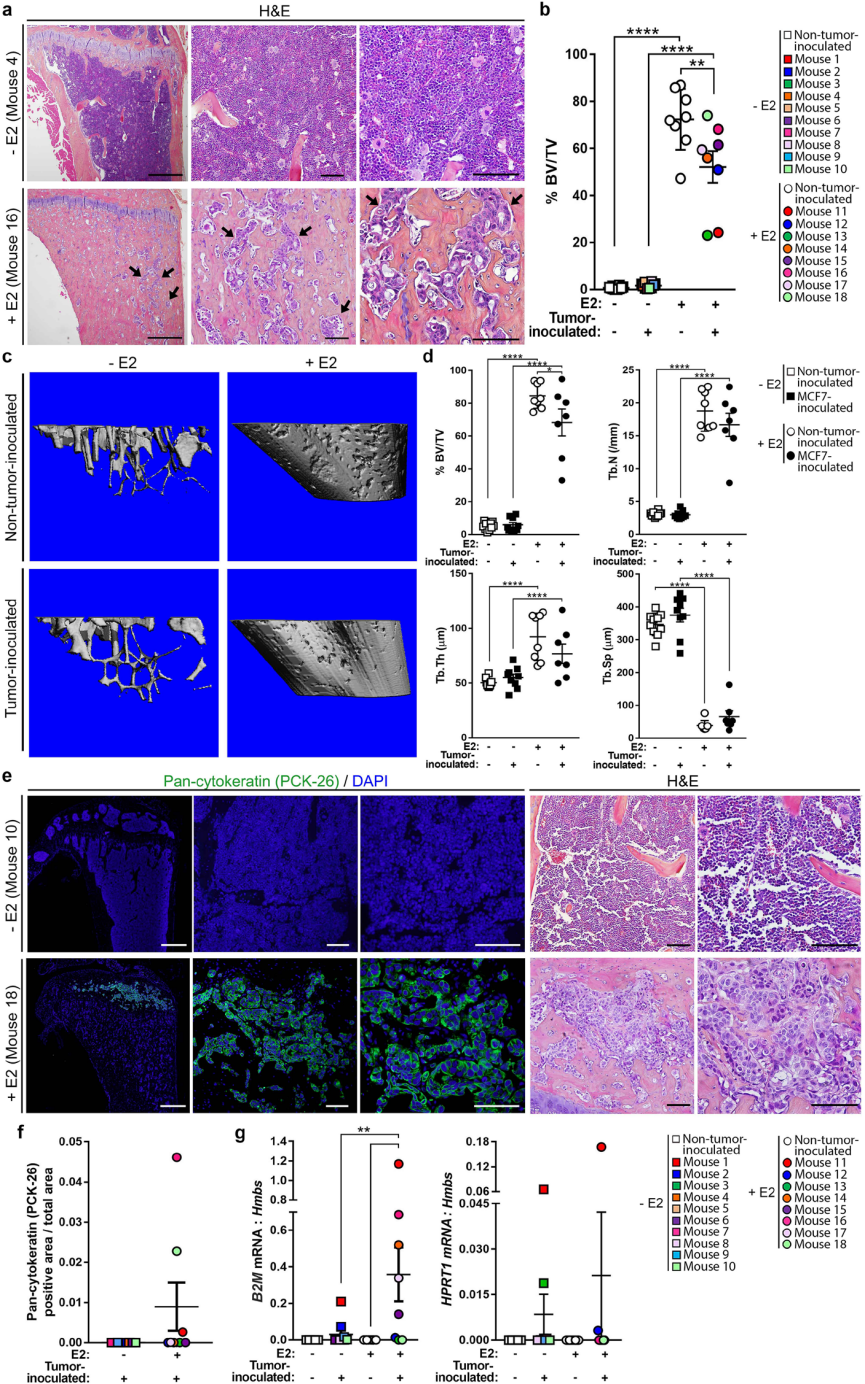
Assessment of MCF7 tumor burden in the bone by histology, immunofluorescence, and qPCR. (**a**) Representative hematoxylin and eosin (H&E) images of tibiae from MCF7-inoculated mice from −E2 (n = 10 mice) and +E2 (n = 8 mice) mice. Arrows indicate tumor cells. Panels left to right = 4X, 20X, 40X of same tibia. Scale bars = 500 μM (left) and 100 μM (right two panels). (**b**) Histomorphometric analysis of bone volume/total volume (%BV/TV) from mice described in (**a**) and non-tumor-inoculated (naïve) mice (n = 10 −E2 mice, n = 8 +E2 mice). (**c**) Representative microCT images of mice described in (**a**,**b**). (**d**) micro-CT analysis of mice described in (**c**). (**e**) Representative images of immunostaining for pan-cytokeratin (PCK-26) and DAPI or H&E from mice described in (**a**). Immunofluorescence panels left to right = 4X, 20X, 40X of same tibia. H&E panels, left = 20X, right = 40X. Scale bars = 500 μM (far left panel) and 100 μM (right four panels). (**f**) Quantitation of pan-cytokeratin (PCK-26) area over total bone area from (**e**). (**g**) qPCR of whole bone homogenate from non-tumor-inoculated (naïve) mice (n = 10 −E2 mice, n = 8 + E2 mice) and MCF7-inoculated mice (n = 10 −E2 mice, n = 8 +E2 mice) for human *B2M* or human *HPRT1* normalized to mouse *Hmbs* (housekeeping gene). (**b**,**d**,**g**) One-way ANOVA with Sidak’s multiple comparisons test, **P < 0.01 and ****P < 0.0001.

Hematoxylin and eosin (H&E) stained tibiae from MCF7-inoculated mice were assessed for the presence of tumor cells based on features including prominent nucleoli, large pale nuclei, increased mitoses, and epithelial morphology. Tumor cells were detected in 2/8 (25%) +E2 mice by our own morphological assessment, but were not detected in any (0/10) −E2 mice compared to non-tumor-inoculated mice (Fig. 3a and Supplementary Fig. 2a). Evaluation of these tibiae by an ACVP board-certified veterinary anatomic pathologist identified tumor cells in two additional +E2 mice resulting in a total of 4/8 (50%) +E2 mice harboring tumor cells in the bone marrow. As previously reported^28^, histomorphometric analysis revealed a significant increase in the average bone volume from ~1% in −E2 mice to ~60% in +E2 mice (Fig. 3a,b) in both non-tumor-inoculated and MCF7-inoculated mice. Further, there was a significant reduction in bone volume in +E2 MCF7-inoculated mice compared to non-tumor-inoculated mice (Fig. 3b). These changes in bone volume were supported by microcomputed tomography (microCT) analysis of a separate cohort of −E2 and +E2 MCF7-inoculated mice (Fig. 3c,d). Further, a significant increase in trabecular number and thickness and a concomitant decrease in trabecular spacing was observed in +E2 compared to −E2 mice, independent of tumor inoculation.

We were unable to find a CD298 antibody that was suitable for immunostaining, and therefore performed pan-cytokeratin staining, which has been previously used to detect neoplastic epithelial cells in the bone marrow of xenograft mouse models and breast cancer patients^3,4,29^. We confirmed cytokeratin expression on MCF7 cells using two independent pan-cytokeratin antibodies (PCK-26 and AE1/AE3) (Supplementary Fig. 2b). The staining pattern was consistent between antibodies and detected tumor cells in 0/10 (0%) −E2 mice and in the same 3/8 (38%) +E2 mice (Fig. 3e,f and Supplementary Fig. 2c). We confirmed that tibiae stained with DAPI alone were not auto-fluorescent in the green channel (Supplementary Fig. 2d). The specificity of the pan-cytokeratin (AE1/AE3) staining was confirmed using adult skin as a positive control and brain and non-tumor-inoculated (naïve) tibiae as negative controls (Supplementary Fig. 2d).

Expression of human beta-2-microglobulin (*B2M*), a human housekeeping gene^17,30^, was detected in bone homogenates from MCF7-inoculated mice in 5/10 (50%) −E2 mice and 5/8 (63%) +E2 mice (Fig. 3g and Supplementary Fig. 3) by qPCR, making this the second most sensitive method of MCF7 tumor cell detection in bone after flow cytometry. qPCR for the human housekeeping gene *HPRT1*^17,30^ was less sensitive but detected tumor cells in 4/10 (40%) −E2 mice and 2/8 (25%) +E2 mice (Fig. 3g and Supplementary Fig. 3).

### Dissemination to bone by murine D2.0R and human SUM159 cells

**Figure 4:**
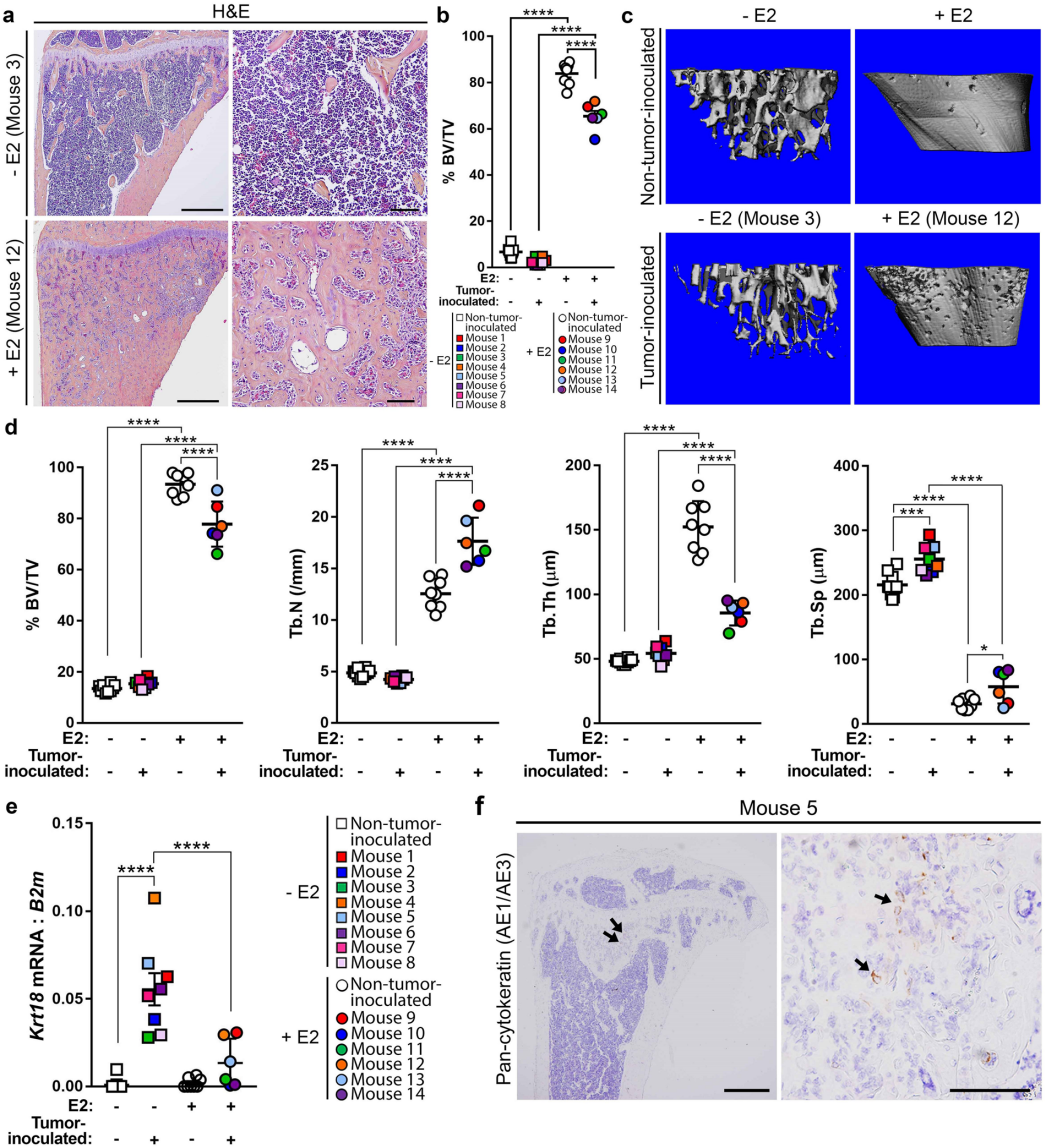
Characterization of D2.0R dissemination to bone. (**a**) Representative hematoxylin and eosin (H&E) images of D2.0R-inoculated tibiae from −E2 (n = 8 mice) and +E2 (n = 6 mice) mice. Left panels = 4X, right panels = 20X of same tibiae. Scale bars = 500 μM (left panel) and 100 μM (right panel). (**b**) Histomorphometric analysis of bone volume/total volume (%BV/TV) from mice described in (**a**) and non-tumor-inoculated (naïve) mice (n = 10 −E2 mice, n = 8 +E2 mice). (**c**) Representative microCT images of mice described in (**a**) and (**b**). (**d**) microCT analysis of mice described in (**c**). (**e**) qPCR analysis of whole bone homogenates from non-tumor-inoculated mice and D2.0R-inoculated mice described in (**a**) and (**b**) for *Krt18*, normalized to mouse *B2m*. (**f**) Positive pan-cytokeratin (AE1/AE3) staining in the tibia from a D2.0R-inoculated mouse (mouse number 5). Arrows indicate cytokeratin-positive tumor cells. Left panel = 4X, right panel = 40X of the same tibia. Scale bars = 500 μM (left panel) and 100 μM (right panel). (**b**,**d**,**e**) One-way ANOVA with Sidak’s multiple comparisons test, *P < 0.05, ***P < 0.001 and ****P < 0.0001.

H&E staining of tibiae from D2.0R-inoculated mice did not reveal any dramatic tumor lesions, irrespective of E2 supplementation compared to non-tumor-inoculated mice (Fig. 4a and Supplementary Fig. 4a). However, assessment of these sections by a veterinary pathologist revealed the presence of tumor cells in 1/9 (11%) −E2 and 2/6 (33%) +E2 mice. Histomorphometric analysis of tibiae from non-tumor-inoculated and D2.0R-inoculated mice revealed a significant increase in bone volume from ~4.5% to ~75% with E2 supplementation (+E2) (Fig. 4b), similar to that observed in the MCF7 model. A significant reduction in bone volume was observed in +E2 D2.0R-inoculated mice compared to non-tumor-inoculated mice (Fig. 4b). To further assess whether bone microarchitecture was altered with D2.0R inoculation and/or E2 supplementation, microCT analysis was performed on −E2 and +E2 non-tumor-inoculated and D2.0R-inoculated mice (Fig. 4c,d). Consistent with histomorphometric analysis of these bones (Fig. 4b), microCT analysis revealed a significant increase in trabecular bone volume and trabecular number and a decrease in trabecular separation in +E2 mice compared to −E2 mice regardless of tumor inoculation (Fig. 4d). Trabecular thickness was significantly greater in +E2 versus −E2 non-tumor-inoculated (naïve) mice (Fig. 4d), but was not statistically different in tumor-inoculated mice. Within the +E2 mice, a significant decrease in bone volume and increase in trabecular separation, with a trend toward a reduction in trabecular thickness, was observed in D2.0R-inoculated mice (Fig. 4d). Surprisingly, there was also a significant increase in trabecular number in these mice (Fig. 4d).

Since the syngeneic D2.0R cell line is of mouse, rather than human origin, cytokeratin 18 (*Krt18*) expression, which is commonly used to identify mammary epithelial cells^31,32^, was used in place of human *B2M* expression following validation of *Krt18* expression in D2.0R cells (Supplementary Fig. 4b). A significant increase in *Krt18* expression was observed in −E2 D2.0R-inoculated mice compared to −E2 non-tumor-inoculated (naïve) mice (Fig. 4e and Supplementary Fig. 5). Interestingly, there was significantly less *Krt18* expression in +E2 D2.0R-inoculated mice compared to −E2 D2.0R-inoculated mice (Fig. 4e). Immunostaining for pan-cytokeratin (AE1/AE3) was detected in a subset of D2.0R cells grown *in vitro* (Supplementary Fig. 4c) and pan-cytokeratin positive tumor cells were detected in the bone marrow of 1/9 (11%) −E2 mice (Fig. 4f).

**Figure 5:**
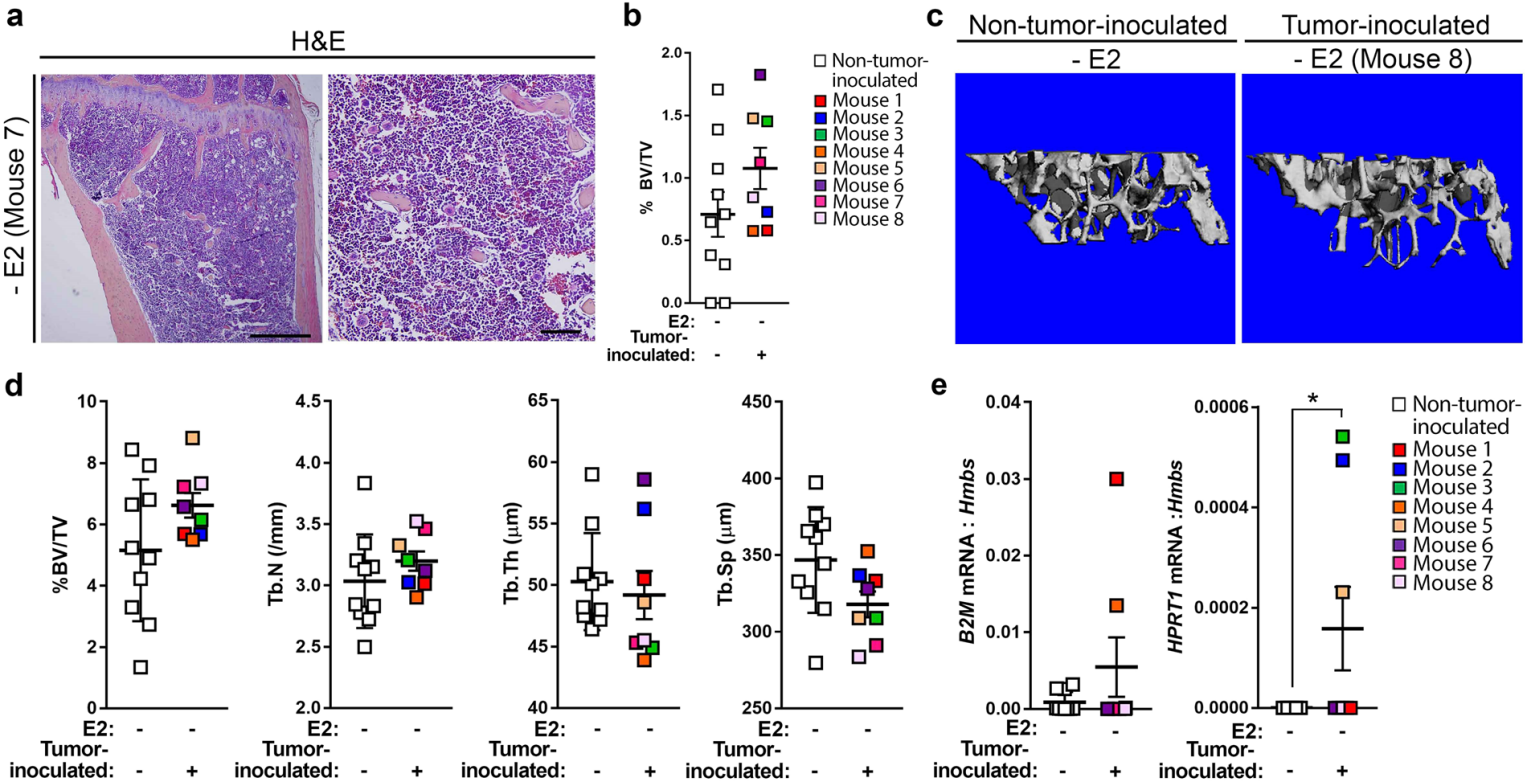
Characterization of SUM159 dissemination to bone. (**a**) Representative H&E images of the tibia from a SUM159-inoculated mouse (n = 8 mice). Left panel = 4X, right panel = 20X of the same tibia. Scale bars = 500 μM (left panel) and 100 μM (right panel). (**b**) Histomorphometric analysis of bone volume/total volume (%BV/TV) from mice described in (**a**). (**c**) Representative microCT images of non-tumor-inoculated (naïve) mice (n = 10 mice) and SUM159-inoculated mice (n = 8 mice). (**d**) micro-CT analysis of mice described in (**c**). (**e**) qPCR of whole bone homogenate from mice described in (**c**) for human *B2M* or human *HPRT1* normalized to mouse *Hmbs* (housekeeping gene). (**e**) Mann-Whitney U-test, *P < 0.05.

H&E staining and morphological assessment of tibiae from SUM159-inoculated mice failed to detect tumor cells in any (0/10) mice compared to non-tumor-inoculated mice (Fig. 5a and Supplementary Fig. 4d), which was confirmed by a veterinary pathologist. Bone microarchitecture was evaluated by histomorphometric analysis (Fig. 5b) and microCT (Fig. 5c,d), which revealed no significant alterations in bone volume or trabecular structure between age-matched non-tumor-inoculated (naïve) and SUM159-inoculated mice. However, consistent with flow cytometric analysis of marrow isolated from SUM159-inoculated mice (Fig. 2f–h), we detected tumor cells by qPCR analysis for human *B2M* expression in 2/8 (25%) mice and human *HPRT1* in 3/8 (38%) mice (Fig. 5e and Supplementary Fig. 6). Similar to D2.0R cells, immunostaining for pan-cytokeratin (AE1/AE3) was detected in a subset of SUM159 cells grown *in vitro* (Supplementary Fig. 4c); however, pan-cytokeratin did not detect tumor cells in the bone marrow of any (0/8) SUM159-inoculated mice (data not shown).

## Discussion

**Figure 6:**
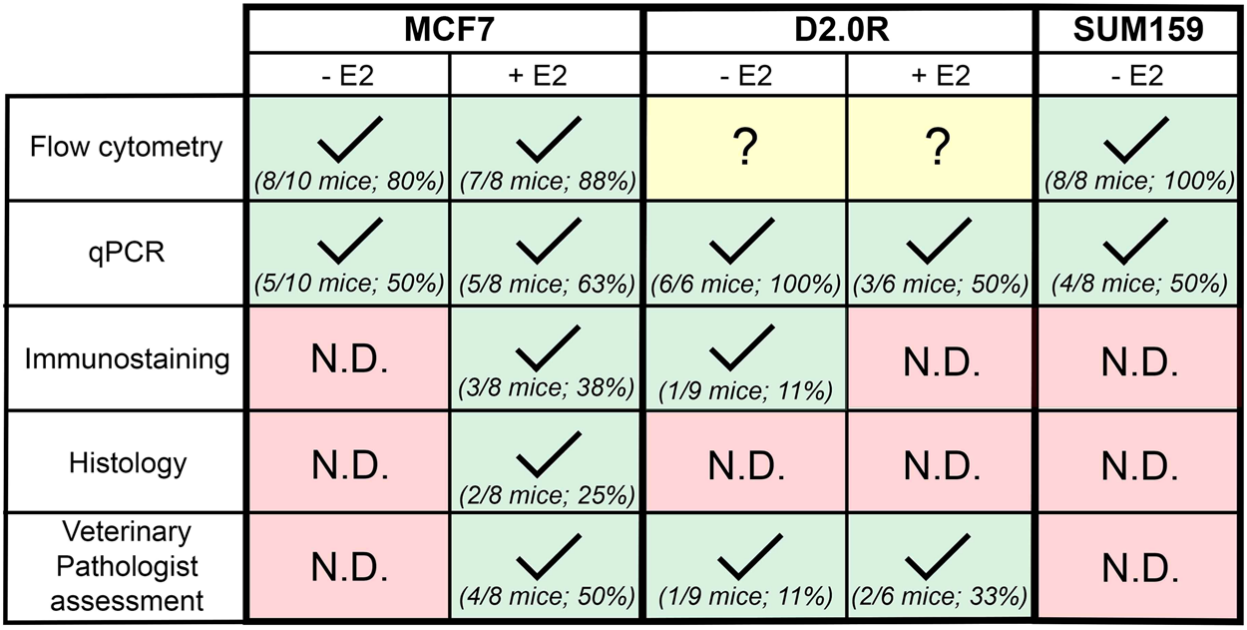
Summary of method efficiency in detecting tumor cells in the bone by model. Check mark indicates positive detection of tumor cells in the bone by the indicated method. N.D. indicates no detectable tumor cells by the given method, despite the use of appropriate positive and negative controls. Question mark indicates the lack of a specific tumor cell marker to detect tumor cells in the bone by the indicated method.

Little is known about the mechanisms that regulate tumor cell homing to the bone marrow and subsequent entry into and exit from dormancy. This is, in part, due to the lack of available *in vivo* models that recapitulate long latency periods observed in humans. Here, we investigated the ability of three different breast carcinoma cell lines (ER+ human MCF7, ER+ murine D2.0R, and ER− human SUM159), to disseminate to the bone following intracardiac inoculation in the presence (+E2) or absence (−E2) of estrogen supplementation. Our data indicate that exogenous estrogen is not required for tumor cell dissemination to the bone marrow in the MCF7 or D2.0R model. However, estrogen is necessary for tumor cells to grow in and colonize the bone marrow in the MCF7 model, since micrometastases detectable by immunostaining were only evident in the +E2 MCF7 model. While MCF7s have been used by multiple groups in bone colonization studies^17,21,33,34^, this is the first report describing the ability of D2.0R and SUM159 cells to home to the bone marrow. The D2.0R cells exhibit a time-course of approximately 7 weeks (similar to the MCF7 model), while the SUM159 cells exhibit an extended latency period (13 weeks), which may be particularly useful for the study of prolonged tumor latency. These groups were sacrificed at the indicated times due to several mice becoming moribund or found deceased due to estrogen toxicities or unknown causes. Thus, the maximum amount of time for the SUM159 model appears to be 13 weeks, since these mice were all −E2 and did not have estradiol toxicity. It also remains unclear whether the D2.0R model, particularly without E2, will spontaneously grow into overt metastases. The data suggest that each of these cell lines home to the bone in >80% of mice (with the exception of the +E2 D2.0R model, which is detected in 50% of mice) as assessed by either flow cytometry, qPCR, or histological assessment, and that different methods of detection are better suited to individual models (Fig. 6). Interestingly, across all models, qPCR is most reliable for detecting tumor burden in bone, but is not as sensitive as flow cytometry, since we have yet to identify an appropriate flow marker for the D2.0R model.

Historically, several types of models have been used to study bone metastasis and colonization with each having their own advantages and limitations. Transgenic mouse mammary carcinoma models such as the MMTV-PyMT model have been used, albeit infrequently, to investigate the spontaneous development of metastases in an immunocompetent mouse^3^. However, these models do not reliably metastasize to the bone and require months to detect disseminated tumor cells in the bone. Thus, intracardiac injection of murine-derived mammary carcinoma cell lines into syngeneic mice or human breast cancer cell lines into immunocompromised mice are used in the majority of bone colonization studies. Since the tumor cells are injected directly into the bloodstream, these are not true bone metastasis models, but rather models of bone colonization. Further, these models are more conducive to genetic manipulation of tumor cells and have a relatively shorter latency period. By far, the murine 4T1 and human MDA-MB-231 are the most commonly used cell lines for tumor-bone studies since they rapidly induce osteolytic bone destruction^16^. Importantly, these models do not recapitulate the latency behavior of DTCs that are thought to occur in breast cancer patients. The SSM2 and SSM3 cell lines derived by the Faccio lab from spontaneous mammary carcinomas in STAT1^−/−^ mice^35^ represent the first mouse ER+ models that consistently form osteolytic lesions in the bone. Although it has not yet been investigated, bone destruction in these models was observed 4–7 weeks after inoculation, suggesting that these cells may lie dormant or proliferate slowly in the bone for some period of time before developing into an overt metastasis. Recently, the dormant bone metastatic (DBM) T47D breast cancer cell derivative was described as a latent tumor model that, similar to the MCF7 model, will eventually develop overt bone metastases with E2 supplementation^36^. These tumor models are useful tools to better understand regulators of DTC dormancy in the bone; however the negative effects of E2 on the bone remains a confounding factor in these models.

In the present study, we observed a significant increase in tumor burden in MCF7 +E2 mice versus −E2 mice by flow cytometry, qPCR, and pan-cytokeratin staining. These results suggest that E2 promotes the growth of MCF7 tumors in the bone, but is not necessary for initial colonization and survival since we detected tumor cells in the bone marrow in 80% of −E2 mice by flow cytometry. These data further suggest that MCF7 cells residing in the bone may lie in a dormant state in the absence of E2, which are confirmatory of reports demonstrating the estrogen dependence of MCF7 parental^21^ and bone-tropic variants^37^ in bone colonization. Importantly, these previous studies relied exclusively on *ex vivo* fluorescence imaging or *in vivo* bioluminescence imaging for the detection of DTCs in the bones of −E2 mice. Similarly, radiographic analysis is classically used to assess bone destruction in tumor models; however, there is no direct correlation with tumor burden given that normal and tumor-induced bone remodeling are indistinguishable by radiography^34^. Therefore, it is critical that other methodologies, especially those that comprehensively analyze the bone marrow, are used to confirm the presence of tumor cells following *in vivo* imaging. This point is evidenced by the presence of osteolytic lesions in the −E2 MCF7 model by radiography at 7 weeks and the detection of rare tumor cells by flow cytometry and qPCR but not histology. Thus, the findings presented herein improve upon the current methods to confirm tumor burden following *in vivo* imaging given our ability to detect and quantify ultra-low tumor burden in the bone using multiple modalities.

Compared to the +E2 MCF7 model, we propose that the −E2 MCF7 model provides a more physiologically relevant system in which to study the timeline of bone colonization, and that flow cytometry, which detected tumor cells in 8/10 (80%) of mice, is the best method to detect bone-disseminated tumor cells in this model (Fig. 6). Importantly, because the −E2 and +E2 MCF7-inoculated mice were sacrificed at the same time point, the question remains whether MCF7 cells in −E2 mice would eventually proliferate into an overt metastasis if the time course was extended. Likewise, it is unknown whether the −E2 D2.0R model would also develop into overt metastases if allowed to continue indefinitely.

Histological assessment by a veterinary pathologist identified a subset of +E2 mice, independent of tumor inoculation, with appreciable endosteal osteosclerosis, myelopthisis, and atypical expansion of mesenchymal cells appearing to be osteoblasts and osteoclasts. Presumably, these manifestations are due to estrogen toxicity as they also appear in non-tumor-inoculated mice (data not shown); however, they can be erroneously identified as tumor cells that have acquired a mesenchymal phenotype. The most extreme cases of this cellular expansion also present as a slight decrease in bone volume as observed for mouse 11 and mouse 13 in Fig. 3b. These observations further demonstrate the importance of confirming the presence of tumor cells by additional methods presented herein besides H&E. MicroCT analysis of estrogen supplemented bones can also prove to be difficult due to the dramatic changes in bone microarchitecture that are observed in +E2 long bones. We observed inconsistencies in bone microarchitecture in the D2.0R model in particular, where there was a significant reduction in bone volume and increase in trabecular spacing, but a paradoxical increase in trabecular number in D2.0R-inoculated versus non-tumor-inoculated (naïve) mice. These results are likely due to the difficulty in contouring the microCT scans as a result of the dramatic increase in bone volume, which can be better appreciated by viewing cross-sections of the tibiae (Supplementary Fig. 7a). It is also possible that the presence of D2.0R cells, even at low numbers, may directly impact bone-resident cells, such that centers of ossification are increased but overall bone volume is significantly reduced.

Importantly, the D2.0R model is advantageous over other tumor models given that the cells are ER+ and are inoculated into immunocompetent mice. Data from several groups suggests that depletion of T cells results in the awakening of dormant tumor cells^38,39^, but the specific role for the immune system in the outgrowth of metastatic tumor cells remains unclear^40^. Thus, the D2.0R model allows for the investigation of the potential impact of the immune system in mediating tumor cell dissemination and colonization of the bone. Because D2.0R cells are of mouse origin, we were unable to use CD298 to detect tumor cells and although we attempted PNA, EpCAM, and mouse cytokeratin staining of these cells *in vitro* (data not shown), we were unable to find a cell marker suitable for flow cytometry that was uniquely expressed on tumor cells and not on mouse bone marrow cells. Surprisingly, a slight reduction in osteolysis was observed over time in the −E2 D2.0R-inoculated mice. These results suggest that D2.0R cells may initially disrupt osteoclast-mediated resorption in the absence of E2 but that this effect diminishes over time. These results are further supported by histomorphometry and microCT analysis of −E2 non-tumor-inoculated (naïve) and D2.0R-inoculated mice at the end point, which revealed no significant difference in bone volume. However, a significant reduction in bone volume was observed in +E2 D2.0R-inoculated mice compared to +E2 non-tumor-inoculated (naïve) mice suggesting that D2.0R cells induce bone loss in the presence of E2. Although tumor burden was enriched for in these mice by *Krt18* expression (Fig. 4e), 4/6 mice did not show evidence of tumor infiltration by pathologic inspection, suggesting that any effects of the tumor cells on the bone microarchitecture are due to changes in bone homeostasis rather than an increase in tumor-induced osteolysis. A significant reduction in *Krt18* expression was observed in +E2 versus −E2 D2.0R-inoculated mice suggesting that, in contrast to the MCF7 model, E2 may not promote tumor growth in the D2.0R model. Additionally, based on the variable pan-cytokeratin staining of D2.0R cells *in vitro* (Supplementary Fig. 4c), we cannot rule out the possibility that E2 alters the cytokeratin expression of inoculated D2.0R cells *in vivo*.

It has been previously reported that SUM159 cells persist as dormant tumor cells in the lung following tail vein injection^24^. Until now, the behavior of these cells in the bone has not been investigated. Using qPCR and flow cytometry, we found that SUM159 cells are detectable in the bone marrow in 50–100% of mice following intracardiac inoculation, and therefore propose the SUM159 cells as a novel human model of ER− breast cancer dissemination to bone. SUM159 cells resemble the claudin-low tumor subtype of breast cancer and thus have reduced expression of epithelial cell adhesion markers and increased stem cell markers including CD44^hi^/CD24^lo^^41^. In addition to pan-cytokeratin, we also attempted to detect SUM159 tumor cells in the bone marrow using CD44, which stained tumor cells *in vitro*, but did not detect any tumor cells *in vivo* (data not shown). Although pan-cytokeratin and CD44 did not reveal any SUM159 cells in the bone, these results do not rule out the possibility that the tumor burden was below the level of detection by immunostaining, particularly since we detected tumor cells by flow cytometry in 100% of SUM159-inoculated mice. Importantly, gene expression profiling of breast cancers suggest that each subtype is a unique disease and that the drivers and effective therapeutics for each subtype may differ^42,43^. Furthermore, patients with ER− breast cancer develop bone metastases at similar rates as those patients with ER+ disease^15^. Thus, the SUM159 model provides a model in which to study factors that regulate homing of tumor cells to the bone or tumor dormancy in a subtype-specific manner.

One limitation of analyzing low tumor burden by immunostaining or H&E is that each histological section represents only a small fraction of the three-dimensional structure of the tibia. In support of this notion, the +E2 MCF7-inoculated mice in which we observed tumor cells by H&E or pan-cytokeratin staining were three of the four mice with the highest number or percentage of CD298+ cells in the bone marrow. Clusters of MCF7 cells were clearly discernible with pan-cytokeratin staining in the bone marrow of +E2 mice, suggesting that the level of tumor burden in the −E2 mice was below the level of immunohistochemical detection. In the D2.0R model, tumor cells heterogeneously expressed pan-cytokeratin (Supplementary Fig. 4c), suggesting that we are likely missing a portion of the tumor cells in the bone marrow using this marker. These conclusions are further supported by the identification of tumor cells in the bone marrow of two +E2 D2.0R-inoculated mice by the veterinary pathologist that were negative for pan-cytokeratin staining. Another source of confusion in the immunohistochemical detection of tumor cells in the bone marrow can be the brown staining of blood pigment, particularly within the synovium and periosteum of the bone (Supplementary Fig. 8) that is observed in both non-tumor-inoculated (naïve) and tumor-inoculated mice. However, cell morphology and the pigment granularity allows for distinction from pan-cytokeratin positive tumor cells.

These data support the superiority of analyzing CD298 expression by flow cytometry, when available, to detect low levels of tumor in the bone over other methods. Additionally, MDA-MB-231 (ER−), T47D (ER+), and human PDX samples have also been shown to express CD298^25^. Analysis of CD298 expression by flow cytometry is a broadly applicable method to investigate tumor burden in the bone following inoculation of various breast cancer cell lines or patient samples. In the context of tumor dormancy, this method can be combined with Hoechst-Pyronin Y staining to distinguish cells that are in a quiescent G_0_ state^44,45^. Furthermore, several groups have reported the use of cell division dyes, such as DiD^46^ or CellTrace Violet^19^, to monitor cell proliferation *in vivo*. Identification of dormant tumor cells at the cellular level *in vivo* remains challenging, in part due to our lack of understanding of whether dormant disseminated tumor cells are truly quiescent or simply growth-restricted^47^; however, in the future these cell division protocols may be optimized in conjunction with CD298 staining to assess cell quiescence. As such, these mouse models may not serve as strict models of tumor dormancy, but do accurately re-capitulate prolonged tumor latency in the bone marrow. In the absence of suitable flow cytometry markers, such as in the D2.0R mammary carcinoma model, qPCR is the second most sensitive method of detection and is recommended for the quantification of tumor burden in the bone marrow. Application of these methods to transgenic models may provide significant advancement to the detection of ultra-low tumor burden in models that do not extensively metastasize to the bone.

In conclusion, our data characterize three distinct models of bone colonization and summarize the most effective methods of detection for each model (Fig. 6). Although the ability to develop into overt metastases has yet to be investigated, these clinically relevant tumor models mimic early tumor dissemination observed in patients during which DTCs survive in the bone marrow for extended periods of time. Further, the ability of flow cytometry or qPCR analysis to detect significant enrichment of low levels of bone-disseminated tumor cells across these cell lines provides a significant advancement to study tumor burden in the bone and illustrates their applicability to future mechanistic studies. These tumor models will allow for the investigation of mechanisms that regulate prolonged latency periods of bone-disseminated tumor cells and for the identification of factors and/or therapeutics that induce a proliferative switch in tumor cells residing in the bone.

## Methods

### Cell lines

Human MCF7 breast cancer cells were obtained from ATCC and murine D2.0R mammary carcinoma cells were a gift from J. Green at the National Cancer Institute. Both cell lines were cultured in DMEM supplemented with 10% fetal bovine serum (FBS) and penicillin/streptomycin (P/S). Human SUM159 breast cancer cells were a gift from the Rutgers Cancer Institute of New Jersey and cultured in Ham’s F12 medium supplemented with 5% FBS, 5 μg ml^−1^ insulin and 1 μg ml^−1^ hydrocortisone as previously described^17^. Human MCF7 and SUM159 cells were recently re-authenticated by ATCC. At this time there is no authentication service available for the D2.0R cell line.

### Animals

All experiments were performed in accordance with the relevant guidelines and regulations of the Animal Welfare Act and the Guide for the Care and Use of Laboratory Animals and were approved by the Institutional Animal Care and use Committee (IACUC) at Vanderbilt University. For the D2.0R +E2 group, 4–6 week old female BALB/c mice (Envigo Corp, Cat #4702) were subcutaneously implanted with a slow-release 17β-estradiol pellet (Innovative Research of America, Cat #SE-121, 0.36 mg/pellet) as previously described^17,37^. Similarly, 4–6 week old athymic nude mice (Jackson, Cat #7850) were implanted with 17β-estradiol pellets for the MCF7 +E2 group. The following day, all mice to be injected with tumor cells were inoculated via intracardiac injection with 1 × 10^5^ tumor cells as previously described^17^ (n = 10 mice injected per group). Mice were euthanized at 7 weeks (D2.0R), 8 weeks (MCF7), or 13 weeks (SUM159 model) post-tumor cell inoculation.

The number of mice indicated in the figure legends (n = 6–10 mice per group) represents the number of mice included in the final analysis. For each cohort, a total of n = 10 mice were originally injected. Mice that (1) died during intracardiac inoculation, (2) became moribund and had to be sacrificed early but had no evidence of tumor burden, or (3) were found deceased, were not included in the analysis. Specifically, for the MCF7 +E2 model, one mouse died during intracardiac inoculation and one mouse was found deceased (final analysis = 8 mice). For the D2.0R +E2 model, one mouse died during intracardiac inoculation and 3 mice were found deceased (final analysis = 6 mice). For the D2.0R −E2 model, two mice were found deceased (final analysis = 8 mice). For the SUM159 model, one mouse died during intracardiac inoculation and one mouse had to be sacrificed early (week 10 of the experiment), but had no evidence of metastatic tumor burden upon gross dissection and examination of radiographs (final analysis = 8 mice). Two +E2 mice in each of the non-tumor-inoculated (naïve) cohorts (athymic nude and Balb/c mice) became moribund and were sacrificed early, presumably due to non-tumor related side effects of estradiol implantation (i.e. bladder stones; final analysis = 8 mice per group).

### Radiography

Radiographic (X-ray) images of mice were obtained as previously described^48^. X-ray images were obtained using a Faxitron LX-60 (34 kV for 8 seconds) and images were quantified using ImageJ software.

### Microcomputed tomography (microCT)

*Ex vivo* microCT was performed on the proximal tibia using the Scanco µCT 50. Scans were initiated from the proximal end of the metaphyseal growth plate and progressed 200 slices distal. Tibiae were scanned at 7 µM voxel resolution, 55-kV voltage, and 200 µA current. Scans were reconstructed and analyzed using the Scanco Medical Imaging Software to determine the bone volume/total volume (BV/TV), trabecular number, thickness, and separation. The most distal slice of the growth plate was used as a reference slice and analysis was set to begin 20 slices distal from this point. A 100 slice region of interest was selected for analysis. For +E2 mice, contours were drawn manually due to the difficulty in distinguishing the cortical bone. For −E2 mice, an automated contouring procedure was applied to separate the trabecular bone from the cortical bone and visually verified for each sample.

### Histology

Hind limbs were dissected and fixed in 10% formalin for 48 hr and decalcified in EDTA (20% pH 7.4) solution for 72 hr. Decalcified bones were embedded in paraffin and 5-μM thick sections were prepared for staining. Hematoxylin and eosin (H&E) staining was performed as previously described^17^. H&E stained sections were analyzed by histomorphometry in the proximal secondary spongiosa using the OsteoMeasure software (Osteometrics, Decatur, GA). Histological analysis of H&E stained tibiae was performed by us as well as an ACVP board-certified veterinary anatomic pathologist who has specific expertise in mouse models of breast cancer. Specifically, tumor cells in the bone were identified based on abnormal features such as prominent nucleoli, increased mitotic rate, large nuclei, high nuclear:cytoplasmic area, epithelial morphology, spindly cells that do not resemble normal bone cells (e.g. osteoblasts), and cells that disrupt the normal architecture of the bone (growth plate, cortical bone).

### Immunostaining

Sections were deparaffinized by heating the slides to 50 °C and placed in Xylene for 5 min and then 3 min. Next, slides were soaked in 100%, 95%, and then 75% ethanol for 3 min each. Slides were slowly changed to deionized water and then rinsed twice in water. The slides were immersed in 10 mM TRIS (pH 9.0) and 1 mM EDTA heated to 150 °C for 20 min. After cooling, slides were rinsed twice with water and then three times with PBS. The deparaffinized sections were blocked in 10% BSA in PBS for 4 hours and incubated with FITC-conjugated primary antibody [Pan-cytokeratin (Sigma; Cat: F0397; 1:50)] in 3% BSA in PBS overnight at 4 °C. The sections were washed three times with 3% BSA in PBS. The coverslips were mounted using VECTASHIELD HardSet Antifade Mounting Medium with DAPI (Vector Laboratories). All images were collected on an Olympus BX41 Microscope equipped with an Olympus DP71 camera using the 4X, 20X, 40X, or 100X plan objectives.

Pan-cytokeratin (AE1/AE3) staining was performed by the Vanderbilt University Medical Center Translational Pathology Shared Resource (TPSR, Nashville, TN) as follows: Slides were placed on the Leica Bond Max IHC stainer. All steps besides dehydration, clearing and coverslipping were performed on the Bond Max. Slides were deparaffinized and enzyme retrieval was performed using Proteinase K (Dako, Carpentinera, CA) for 5 minutes. Slides were placed in a Protein Block (Ref# x0909, DAKO) for 10 minutes. The sections were incubated with Cytokeratin (Catalog-Z0622, Dako) diluted 1:4,000 for one hour. The Bond Refine Polymer detection system was used for visualization and slides were then dehydrated, cleared and coverslipped.

### Flow cytometry

One hindlimb was flushed using centrifugation (−E2 mice) or crushed using a mortar and pestle (+E2 mice) to obtain the bone marrow. The bone marrow was filtered through a 40 μm cell strainer to separate the cells from bone debris. Cells were suspended in red blood cell lysis buffer for 5 minutes on ice, spun down, and washed twice with PBS. Bone marrow (1 × 10^6^ cells) was stained in 100ul of 1% BSA in PBS with 175 ng CD298 antibody (BioLegend, Cat #341704) for 30 minutes on ice in the dark. Cells were washed with PBS and resuspended with 1% BSA in PBS and 25 ng Propidium Iodide (BD Pharmingen, 556463). Flow cytometry experiments were analyzed in the VMC Flow Cytometry Shared Resource using the 5-laser BD LSRII. Datasets were analyzed using FlowJo software (FlowJo, LLC). Cells were gated based on forward scatter and side scatter and then live cells (PI-) were gated using PE-CD298 stained bone marrow as a fluorescence minus one negative control. The dead cells (PI + ) were gated out and are not included in the representative plots shown in the figures.

### Real-time PCR

Intact femora were homogenized in 1 mL TRIzol (Life Technologies), spun down to clear the lysate, phenol-chloroform extracted, DNase digested (TURBO DNA-free Kit, Life Technologies), and cDNA synthesized (1ug RNA, iScript cDNA Synthesis Kit, Bio-Rad) per the manufacturer’s instructions. Real-time PCR was performed using iTaq^TM^ Universal SYBR Green Supermix (Bio-Rad) on a QuantStudio 5 (Thermo Fisher) with the following conditions: 2 min at 50 °C, 10 min at 95 °C, (15 s at 95 °C, 1 min 60 °C) x40 cycles followed by dissociation curve (15 s 95 °C, 1 min 60 °C, 15 s 95 °C). For each biological replicate, three technical replicates were performed for each gene analyzed. If only one technical replicate had detectable expression then the sample was considered negative. Non-template controls and non-tumor-inoculated mice were included as negative controls for each gene analyzed. Analysis was performed by normalizing the expression of the target gene (*B2M*, *HPRT1*, *Krt18*) to the average *Hmbs* expression within the same sample to determine ΔC_t_. The ΔC_t_ was transformed (2^−ΔCt^) and the average of the three technical replicates was calculated. The average 2^−ΔCt^ for each mouse is presented as target gene “(*B2M*, *HPRT1*, *Krt18*) mRNA: *Hmbs”* in the figures. Human primers for beta-2-microglobulin (*B2M*) and hypoxanthine phosphoribosyltransferase 1 (*HPRT1*) were previously published^17^. Mouse primers for hydroxymethylbilane synthase (*Hmbs)* were previously published^49^. *Krt18* primers were designed using PrimerBlast (NCBI) against the mouse genome (*Mus musculus*) and validated by dissociation: *Krt18* (F-TGCCAGCTCTG GATTGACTG, R-GTTCCTCGCGGTTCTTCTGA).

### Statistical methods

For all studies, *n* per group is as indicated in the figure legend and the scatter dot plots indicate the mean of each group and the standard error of the mean. All graphs and statistical analyses were generated using Prism software (Graphpad). All *in vitro* and *in vivo* assays were analyzed for statistical significance using Mann-Whitney U-test or ANOVA with Sidak’s multiple comparisons test. For all analyses P < 0.05 was statistically significant, and *P < 0.05, **P < 0.01, ***P < 0.001, ****P < 0.0001.

## Electronic supplementary material

Supplementary Data

Supplementary Figure 3

Supplementary Figure 5

Supplementary Figure 6

## Data Availability

The data that support the findings of this study are available from the corresponding author upon reasonable request.
