# Supplementary material for: Enrichment and detection of bone disseminated tumor cells in models of low tumor burden
Source: Sci Rep. 2018 Sep 24;8:14299. doi: 10.1038/s41598-018-32653-2 (PMC6155169; doi:10.1038/s41598-018-32653-2)
Supplement: Supplementary file 1 — Supplementary Data [file 41598_2018_32653_MOESM1_ESM.pdf]

Enrichment and detection of bone disseminated tumor cells in models of low tumor burden

Miranda E. Sowder[1,2] and Rachelle W. Johnson*[1,2,3]

[1]Program in Cancer Biology, Vanderbilt University, Nashville, TN, 37232

[2]Vanderbilt Center for Bone Biology, Department of Medicine, Division of Clinical Pharmacology, Vanderbilt University Medical Center, Nashville, TN 37232

[3]Department of Medicine, Division of Clinical Pharmacology, Vanderbilt University Medical Center, Nashville, TN, 37232

*Corresponding author:

Rachelle W. Johnson

2215b Garland Ave

1165C Medical Research Building IV

Vanderbilt University

Nashville, TN 37232

Email: rachelle.johnson@vanderbilt.edu

Phone: 615-875-8965

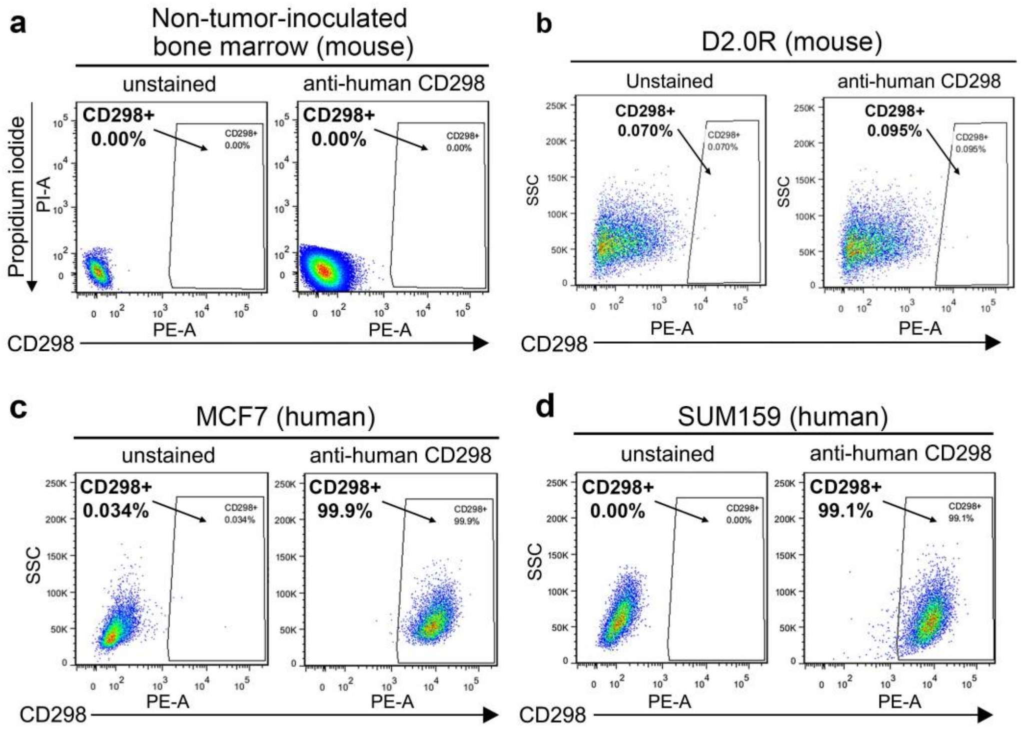

Supplementary Figure 1. CD298 expression in non-tumor-bearing bone marrow and cells grown *in vitro*. (a) Representative flow cytometry plot of CD298 staining in non-tumor-inoculated (naïve) mouse bone marrow (representative of n=3 mice). (b) Flow cytometry plot of CD298 staining in (b) mouse D2.0R, (c) human MCF7, and (d) human SUM159 cells grown *in vitro* (n=1 experiment).

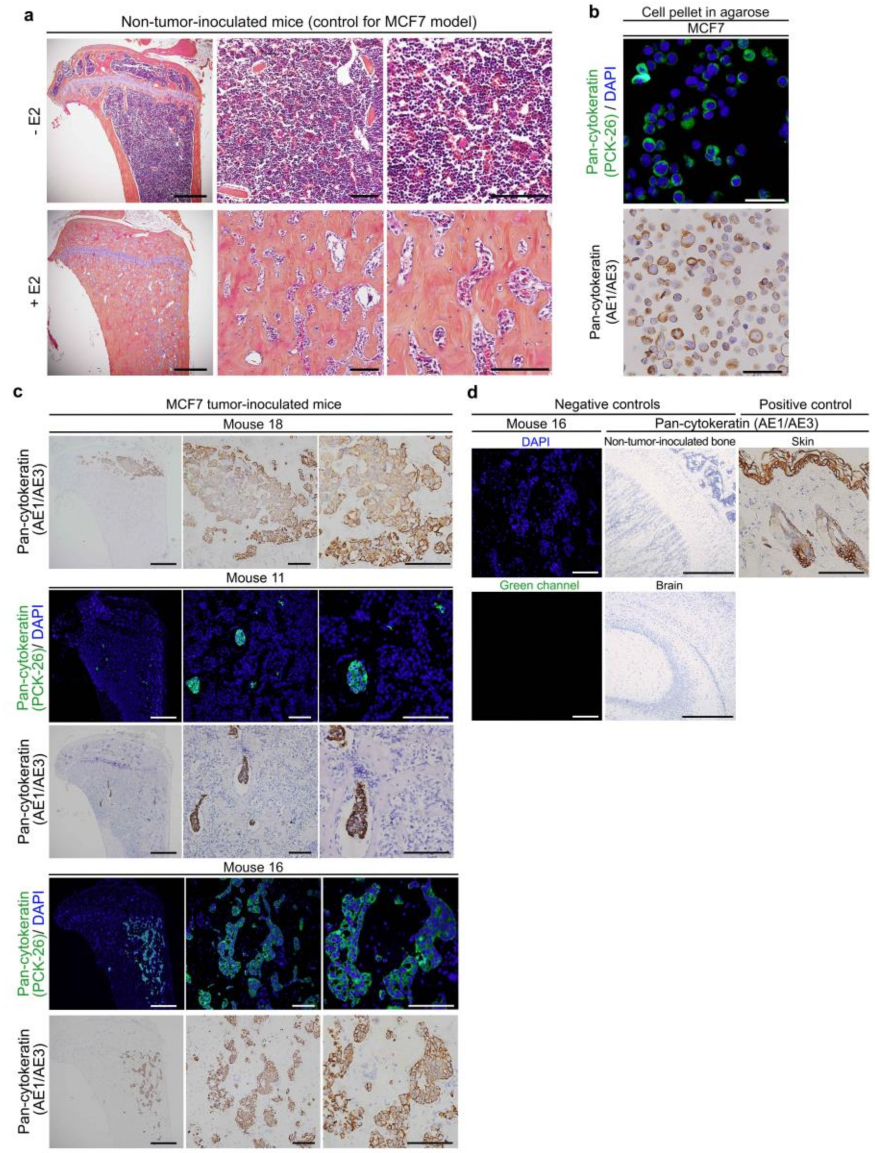

Supplementary Figure 2. Immunostaining for pan-cytokeratin in tumor cells and bones of tumor-inoculated mice. (a) Representative hematoxylin and eosin (H&E) images of tibiae from non-tumor-inoculated -E2 (n=10) and +E2 (n=8) mice. Panels left to right = 4X, 20X, 40X of same tibia. Scale bars = 500μM (left) and 100μM (right two panels). (b) Representative images of pan-cytokeratin staining (PCK-26 and AE1/AE3) in MCF7 cells grown *in vitro* and prepared as a cell pellet in agarose for sectioning and staining (n=1 experiment). Scale bars = 100μM. (c) Positive immunostaining for pan-cytokeratin (PCK-26 and AE1/AE3) in the tibiae of three different MCF7-inoculated mice. Panels left to right = 4X, 20X, 40X of same tibia. Scale bars = 500μM (left panel) and 100μM (right two panels). (d) Representative images of tibiae stained with DAPI alone showing no autofluorescence in the green channel and non-tumor-bearing (naïve) tibiae or brain stained with pan-cytokeratin (AE1/AE3) as negative controls. Skin stained with pan-cytokeratin (AE1/AE3) was used as a positive control. Scale bars = 100μM (left panels, top and bottom) and 500μM (right two panels, top and bottom).

Supplementary Figure 3. Raw data for qPCR analysis of MCF7 model. Raw Ct values and deltaCt analysis of technical and biological replicates for hydroxymethylbilane synthase (*Hmbs*), hypoxanthine phosphoribosyltransferase 1 (*HPRT1*), and beta-2-microglobulin (*B2M*).

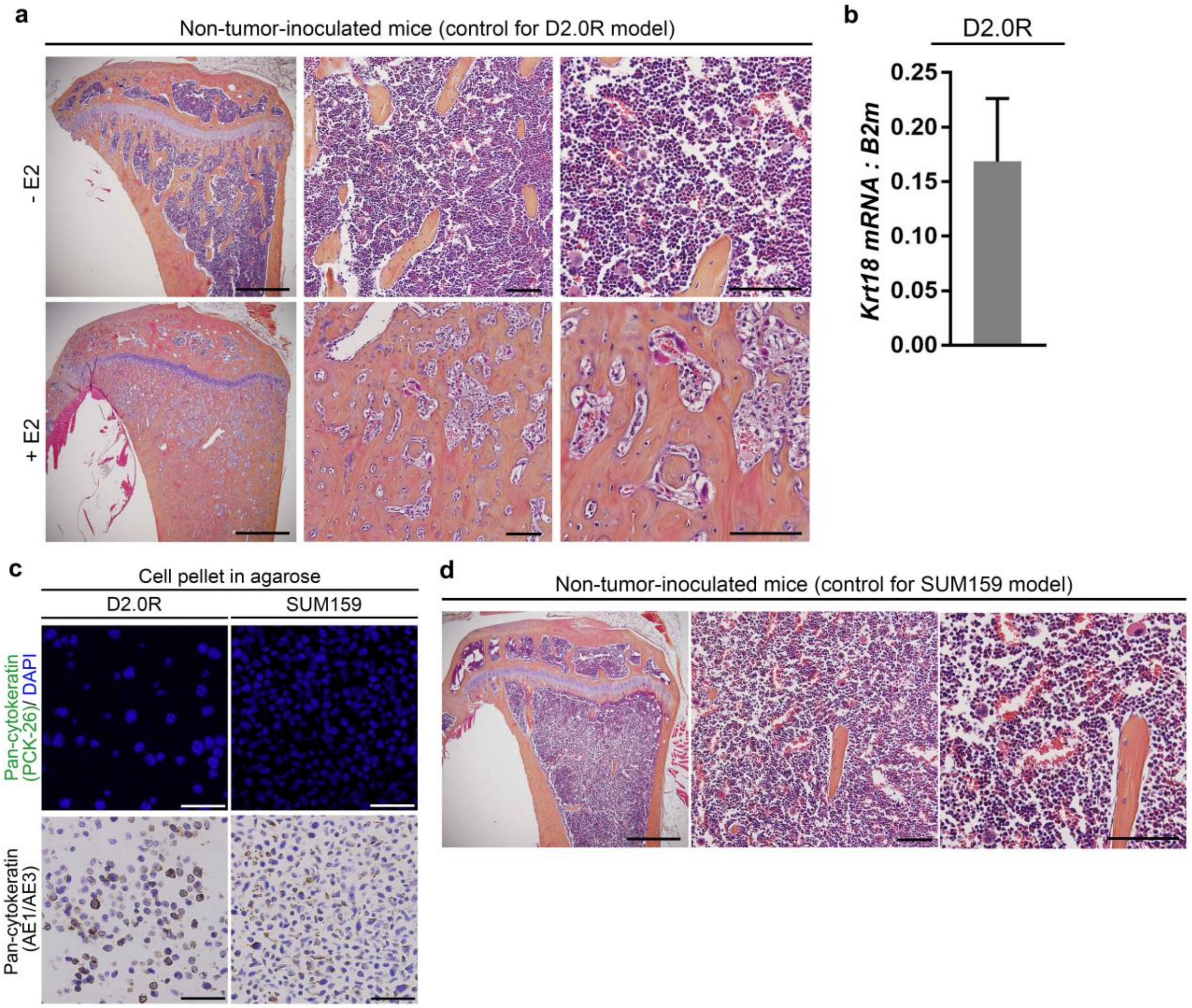

Supplementary Figure 4. H&E images of non-tumor-inoculated mice for D2.0R and SUM159 models and immunostaining for pan-cytokeratin in D2.0R and SUM159 cells grown *in vitro.* (a) Representative hematoxylin and eosin (H&E) images of tibiae from non-tumor-inoculated -E2 (n=10) and +E2 (n=8) mice as controls for the D2.0R model. Panels left to right = 4X, 20X, 40X of same tibia. Scale bars = 500μM (left) and 100μM (right two panels). (b) Expression of *Krt18* in D2.0R cells grown *in vitro* (n=3 replicates from 3 experiments). (c) Pan-cytokeratin (PCK-26 and AE1/AE3) staining of D2.0R and SUM159 cells grown *in vitro* and prepared as cell pellets in agarose for sectioning and staining. (d) Representative hematoxylin and eosin (H&E) images of tibiae from non-tumor-inoculated (n=10) mice as controls for the SUM159 model. Panels left to right = 4X, 20X, 40X of same tibia. Scale bars = 500μM (left) and 100μM (right two panels).

Supplementary Figure 5. Raw data for qPCR analysis of D2.0R model. Raw Ct values and deltaCt analysis of technical and biological replicates for hydroxymethylbilane synthase (*Hmbs*) and keratin 18 (*Krt18*).

Supplementary Figure 6. Raw data for qPCR analysis of SUM159 model. Raw Ct values and deltaCt analysis of technical and biological replicates for hydroxymethylbilane synthase (*Hmbs*), hypoxanthine phosphoribosyltransferase 1 (*HPRT1*), and beta-2-microglobulin (*B2M*).

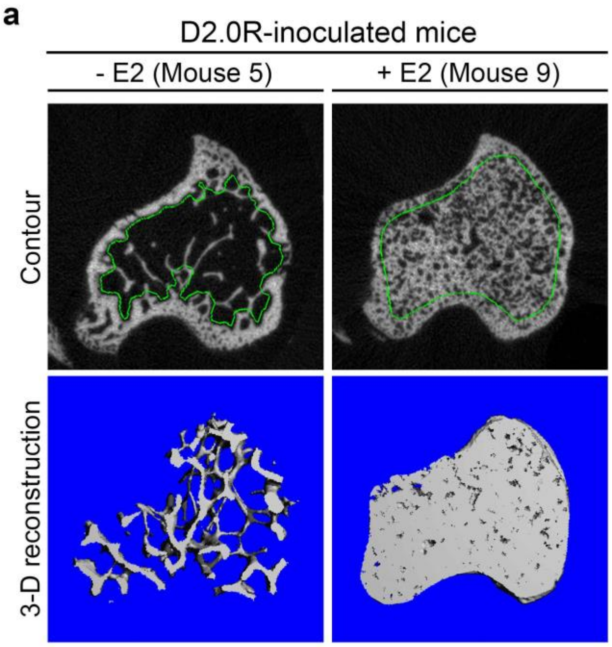

Supplementary Figure 7. microCT contours and 3-D reconstruction for D2.0R model. Representative images of drawn microCT contours (green line) and corresponding 3-D reconstruction for -E2 (n=8 mice) and +E2 (n=6 mice) D2.0R-inoculated mice.

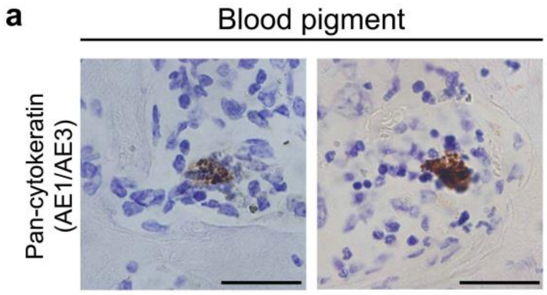

Supplementary Figure 8. Non-specific staining for cytokeratin in the bone marrow. Blood pigment present in pan-cytokeratin (AE1/AE3) stained tibiae. Scale bars = 25µM.
